# The Mechanism of Short-Term Monocular Pattern Deprivation-Induced Perceptual Eye Dominance Plasticity

**DOI:** 10.3389/fnhum.2022.854003

**Published:** 2022-05-31

**Authors:** Jiayu Tao, Zhijie Yang, Jinwei Li, Zhenhui Cheng, Jing Li, Jinfeng Huang, Di Wu, Pan Zhang

**Affiliations:** ^1^Department of Psychology, Chengde Medical University, Chengde, China; ^2^Department of Psychology, Hebei Normal University, Shijiazhuang, China; ^3^Department of Medical Psychology, Air Force Medical University, Xi'an, China

**Keywords:** monocular pattern deprivation, perceptual eye dominance, binocular phase combination, binocular contrast combination, plasticity, MCM

## Abstract

Previously published studies have reported that 150 min of short-term monocular deprivation temporarily changes perceptual eye dominance. However, the possible mechanisms underlying monocular deprivation-induced perceptual eye dominance plasticity remain unclear. Using a binocular phase and contrast co-measurement task and a multi-pathway contrast-gain control model (MCM), we studied the effect of 150 min of monocular pattern deprivation (MPD) in normal adult subjects. The perceived phase and contrast varied significantly with the interocular contrast ratio, and after MPD, the patched eye (PE) became dominant. Most importantly, we focused on the potential mechanisms of the deprivation effect. The data of an averaged subject was best fitted by a model, which assumed a monocular signal enhancement of the PE after the MPD. The present findings might have important implications for investigations of binocular vision in both normal and amblyopic populations.

## Introduction

In recent years, several studies have revealed that following a short period of patching of one eye of adult observers, this eye is temporarily weighted more than the fellow eye in binocular perception (Lunghi et al., 2011; Zhou et al., 2013a, 2014, 2017a,b; Lunghi and Sale, 2015; Zhou and Hess, 2016; Bai et al., 2017; Kim et al., 2017; Spiegel et al., 2017; Wang et al., 2017; Yao et al., 2017; Baldwin and Hess, 2018; Ding et al., 2018; Min et al., 2018, 2019; Ramamurthy and Blaser, 2018; Finn et al., 2019; Sheynin et al., 2019). Lunghi et al. (2011) first found this phenomenon using a short 150-min period of monocular pattern deprivation (MPD). The shift in perceptual eye dominance is temporary. The peak effect is observed immediately after patch removal and then decreases gradually, lasting ≈30–90 min (Lunghi et al., 2011; Zhou et al., 2013a; Min et al., 2018).

The magnitude and duration of this phenomenon are mainly affected by the variables of occlusion duration, occlusion form, and measurement task. First, for occlusion duration, although some studies have demonstrated monocular deprivation effects with 15 min of occlusion (Kim et al., 2017; Min et al., 2018), typically, most studies of short periods of monocular deprivation in normal adults found stable occlusion effects with 150 min of occlusion (Lunghi et al., 2011; Zhou et al., 2013a, 2017b; Bai et al., 2017; Wang et al., 2017; Yao et al., 2017; Baldwin and Hess, 2018). Second, the occlusion effect can be elicited by a translucent patch (also called a diffuser, which excludes pattern information and blocks about 20% luminance information) (Lunghi et al., 2011; Zhou et al., 2013a; Baldwin and Hess, 2018; Min et al., 2018, 2019), by an opaque patch (also called light-tight, which removes both pattern and luminance information) (Zhou et al., 2013a), a dichoptic movie (which shows different information to each eye) (Zhou et al., 2014), a neutral-density filter patch (Zhou and Hess, 2016; Yao et al., 2017), or a kaleidoscope patch (Ramamurthy and Blaser, 2018). Among the different occlusion methods, translucent occlusion has been adopted most frequently and is much more convenient. Zhou et al. (2013a) have shown similar effects (magnitude and duration) for a translucent patch and an opaque patch, suggesting that the monocular occlusion effects are dependent on the interocular differences of pattern information. Furthermore, these monocular deprivation effects have been shown in both binocular combinations (Zhou et al., 2013a, 2014, 2017a,b; Wang et al., 2017; Yao et al., 2017; Min et al., 2018, 2019) and binocular rivalry (competition) (Lunghi et al., 2011; Lunghi and Sale, 2015; Bai et al., 2017; Kim et al., 2017; Finn et al., 2019) tasks. Min et al. (2019) have suggested that, from an ecological perspective, the binocular combination task is a more typical input than the binocular rivalry task. Electrophysiology and brain imaging studies have reported that the deprivation effect involves the early visual cortex (Lunghi et al., 2015a,b; Zhou et al., 2015; Binda et al., 2018). However, the possible mechanism of short-term monocular deprivation-induced perceptual eye dominance alterations in normal adults remains unclear.

**Figure 1:**
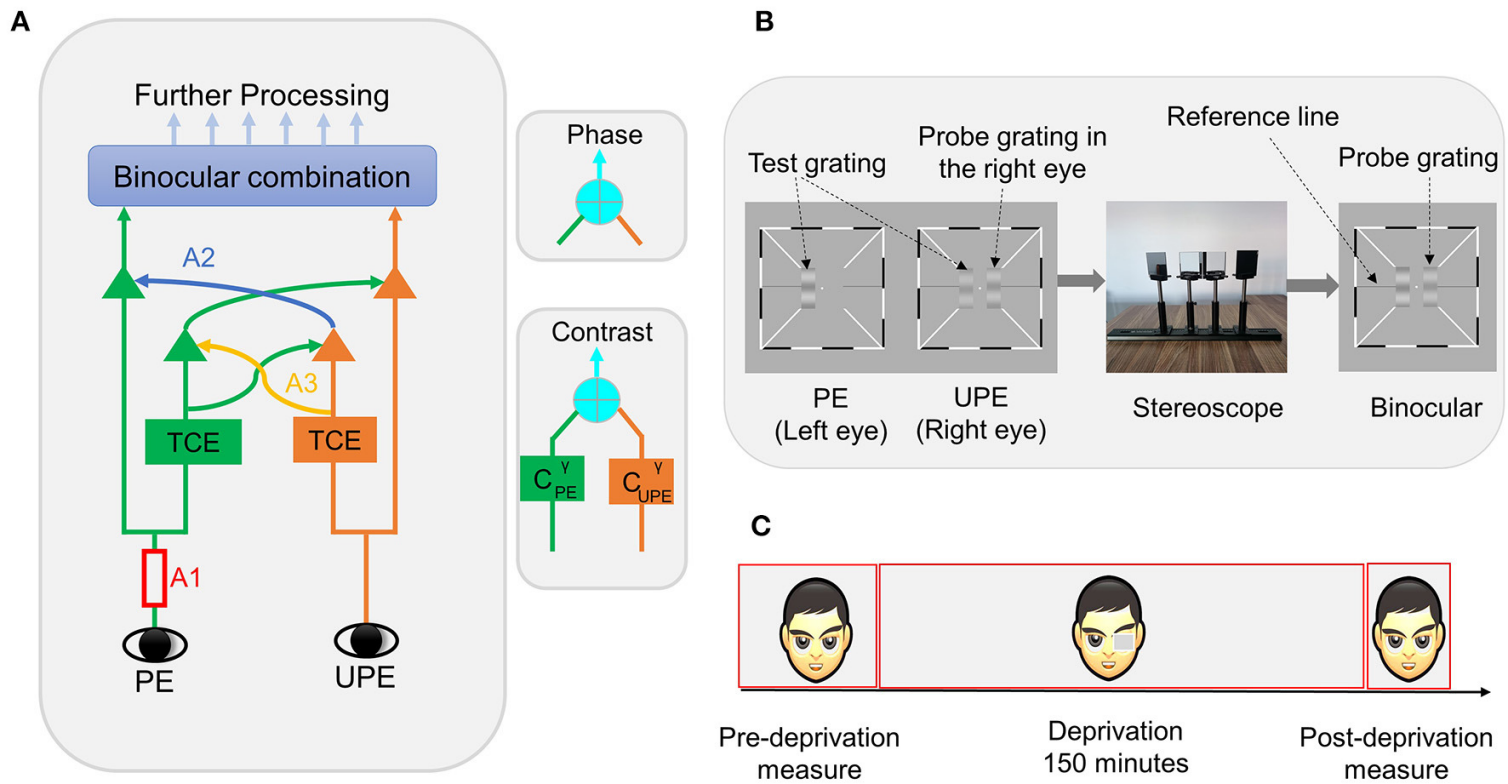
**(A)** Schematic diagram of the binocular combination of the MCM. PE represents the patched eye, and UPE represents the unpatched eye; A1 represents the monocular signal, which first goes through double interocular contrast-gain control; TCE represents total contrast energy; A2 represents the exertion of gain control by each eye onto the other eye's signal in proportion to its signal contrast energy, and A3 represents the exertion of gain control by each eye over that of the other eye. The phase and contrast of the cyclopean images are computed in separate pathways. **(B)** Binocular phase and contrast combination test. Three sine-wave gratings are presented dichoptically. The two test gratings are fused into one standard grating by a stereoscope. Subjects are tasked with adjusting both the phase and contrast of the grating to match those of the standard grating. **(C)** Experimental design. Subjects perform phase and contrast co-measurement tasks at different times.

In recent years, a new multi-pathway contrast-gain control model (MCM) has been developed. The MCM extends other models (Ding and Sperling, 2006; Meese et al., 2006; Baker et al., 2007; Georgeson et al., 2016) by explicitly considering both the contrast and phase in binocular combinations. The MCM is an effective tool for measuring monocular and interocular mechanisms in normal and amblyopic populations (Huang et al., 2010, 2011). Researchers have used the MCM to model the sophisticated data pattern of human adult binocular phase and contrast combinations and found that at least two independent pathways are involved (Huang et al., 2010). More importantly, Huang et al. (2011) successfully used six parameters to model the relationship between perceived phase and contrast of the cyclopean image and interocular contrast ratios in binocular combinations (see Figure 1A and Modeling section) and revealed that the mechanisms of amblyopia include monocular (attenuation of the amblyopic eye signal) and interocular deficits (stronger direct and indirect interocular inhibition). This study indicated that the MCM could distinguish the potential involvement of one or any combination of three mechanisms [signal gain in the non-dominant eye (A1), direct interocular inhibition (A2), and indirect interocular inhibition (A3)] that induced imbalance between the eyes in amblyopia. In other words, therefore, the MCM is also a potentially useful tool for analyzing the mechanism underlying a certain experimental treatment that causes changes in perceptual eye dominance.

The aim of our work was to use MCM to explain the underlying mechanism of changes in perceptual eye dominance caused by 150 min of MPD in normal adults. We focused on the potential involvement of one or any combination of three mechanisms for the deprivation effect. According to the findings from existing research, we hypothesize that monocular deprivation through monocular and/or interocular mechanisms affects binocular function.

## Methods

### Participants

Nine adult participants with corrected visual acuity of both eyes ≥1.0 were recruited. The average age of the participants was 19.6 ± 0.97 (mean ± *SD*). All participants signed informed written consent before the experiment. The procedures of the current study were approved by the Ethical Committee of Chengde Medical University and followed the Declaration of Helsinki.

### Apparatus

The stimuli were produced by a desktop PC running MATLAB with PsychToolbox-3 (Pelli, 1997) and were presented on an Asus monitor with a 120 Hz refresh rate and 1,920 × 1,080 resolution. A stereoscope was used to help generate a single cyclopean image. The distance from the subject's eyes to the screen was 141 cm.

### Stimuli and Procedure

In the phase and contrast co-measurement task, subjects were presented with a white fixation cross (0.11° × 0.11°) in the center of the screen and high-contrast black and white frames (0.11° × 6°) with diagonal bars (0.11° × 2.33°) to both eyes in each trial. After reaching the correct vergence, the subject was asked to press the space bar to begin the task procedure, and three horizontal sine-wave gratings (0.67° × 2°) (Figure 1B) were displayed, including two test gratings and a probe grating. The procedure randomly set the initial phase and contrast values of the probe grating. The two test gratings' luminance profiles as viewed by each eye were as follows:


(1) $$\begin{array}{l} Lum_{PE}\left(y\right)=L_{0}\left[1-C_{0}cos\left(2\pi fy\pm \frac{\theta }{2}\right)\right] \end{array}$$

(2) $$\begin{array}{l} Lum_{UPE}\left(y\right)=L_{0}\left[1-\delta C_{0}cos\left(2\pi fy\mp \frac{\theta }{2}\right)\right] \end{array}$$

where PE represents the patched eye, UPE represents the unpatched eye, *L*_0_ represents the value of the background in grayscale, the sine-wave grating spatial frequency is ƒ = 1 *c*/deg, the six interocular contrast ratios (δ*)* are 0, 0.2, 0.4, 0.6, 0.8, and 1, and the base contrast (*C*_0_) is 0.32. The two gratings, which differed by a 45° (θ) phase shift, were monitored by a stereoscope to produce a single fusion grating.

The probe grating luminance profile, which is only presented in one eye, was determined using the following formula:


(3) $$\begin{array}{l} Lum_{p}\left(y\right)=L_{0}\left[1-C_{p}cos\left(2\pi fy+\theta_{p}\right)\right] \end{array}$$

where the probe grating spatial frequency is identical to that of the test gratings (ƒ = 1 *c*/deg). The subjects were asked to adjust both the phase (θ_p_) and contrast (*C*_p_) of the probe grating to match those of the cyclopean image (standard grating).

Subjects could freely adjust each dimension until they were satisfied in both dimensions. To help subjects easily fuse the images, the high-contrast frames stayed on the display screen during the task.

### Design

This study adopted a within-subjects design. The experiment is divided into three stages (Figure 1C): (1) the pre-deprivation stage: the subject was asked to complete the full phase and contrast co-measurement task over eight blocks. For each block, one base contrast (*C*_0_ = 0.32), six interocular ratios, one phase difference (Δθ = 45°), two configurations, and two probe eyes were measured. The full task was measured over a total of 192 (eight blocks × two configurations × two probe eye conditions × six interocular contrast ratios) trials; (2) the deprivation stage: the non-dominant eye was patched for 150 min using a translucent eye-patch; (3) the post-deprivation stage: lasting ~10 min, in this stage, the subjects were asked to complete a brief phase and contrast co-measurement task spanning only three blocks and three interocular contrast ratio conditions (δ = 0, 0.8, 1). The fast task was measured over a total of 36 (three blocks × two configurations × two probe eye conditions × three interocular contrast ratios) trials. The subjects could freely perform common tasks such as reading and walking during the deprivation period. Before the formal experiment, the subject was asked to practice the test with hundreds of trials to ensure that they could complete the task quickly and well. We determined the non-dominant eye using the phase and contrast co-measurement task data obtained from the practice stage.

## Results

To evaluate the effect of deprivation on the perceived phase, we performed a 2 (deprivation stage: pre, post) × 3 (interocular contrast ratio: 0, 0.8, and 1) within-subject repeated-measures ANOVA. As expected, the main effect of interocular ratio [*F*_(2, 16)_ = 187.77, *p* < 0.001] and the main effect of deprivation stage [*F*_(1, 8)_ = 68.78, *p* < 0.001] were both significant. The interaction effect between the two factors was also significant [*F*_(2, 16)_ = 12.14, *p* < 0.001]. *Post-hoc* tests (least significant difference, LSD) showed that the perceived phase during the deprivation stage condition differed at two interocular contrast ratios, 0.8 (pre, mean ± SE = 1.56 ± 1.91; post, mean ± SE = 12.89 ± 2.84, *p* = 0.001) and 1 (pre, mean ± SE = −4.86 ± 2.54; post, mean ± SE = 5.56 ± 3.01, *p* < 0.001) but not at an interocular contrast ratio of 0 (*p* = 0.47).

Then, we performed a 2 (deprivation stage: pre, post) × 3 (interocular contrast ratio: 0, 0.8, and 1) × 2 (probe eye: PE and UPE) repeated-measures ANOVA to evaluate the effect of patching on perceived contrast. We first normalized perceived


(4) $$\begin{array}{l} \theta^{\prime }=2tan^{-1}\left[\frac{\frac{A_{1}C_{0}+\rho A_{1}^{1+\gamma_{1}}C_{0}^{1+\gamma_{1}}}{1+\rho A_{1}^{\gamma_{1}}C_{0}^{\gamma_{1}}+A_{2}\rho \delta^{\gamma_{1}}C_{0}^{\gamma_{1}}}-\frac{\delta C_{0}+A_{3}\rho \delta^{1+\gamma_{1}}C_{0}^{1+\gamma_{1}}}{1+\rho A_{1}^{\gamma_{1}}C_{0}^{\gamma_{1}}+A_{3}\rho \delta^{\gamma_{1}}C_{0}^{\gamma_{1}}}}{\frac{A_{1}C_{0}+\rho A_{1}^{1+r_{1}}C_{0}^{1+r_{1}}}{1+\rho A_{1}^{\gamma_{1}}C_{0}^{\gamma_{1}}+A_{2}\rho \delta^{\gamma_{1}}C_{0}^{\gamma_{1}}}+\frac{\delta C_{0}+A_{3}\rho \delta^{1+\gamma_{1}}C_{0}^{1+\gamma_{1}}}{1+\rho A_{1}^{\gamma_{1}}C_{0}^{\gamma_{1}}+A_{3}\rho \delta^{\gamma_{1}}C_{0}^{\gamma_{1}}}}tan\left(\frac{\theta }{2}\right)\right], \end{array}$$

(5) $$\begin{array}{l} C^{\prime }={\left[{\left(\frac{A_{1}C_{0}+\rho A_{1}^{1+\gamma_{1}}C_{0}^{1+\gamma_{1}}}{1+\rho A_{1}^{\gamma_{1}}C_{0}^{\gamma_{1}}+A_{2}\rho \delta^{\gamma_{1}}C_{0}^{\gamma_{1}}}\right)}^{\gamma_{2}}+{\left(\frac{\delta C_{0}+A_{3}\rho \delta^{1+\gamma_{1}}C_{0}^{1+\gamma_{1}}}{1+\rho A_{1}^{\gamma_{1}}C_{0}^{\gamma_{1}}+A_{3}\rho \delta^{\gamma_{1}}C_{0}^{\gamma_{1}}}\right)}^{\gamma_{2}}\right]}^{\frac{1}{\gamma_{2}}}, \end{array}$$

contrast, which differed between subjects, by dividing it in different interocular contrast ratios by perceived contrast when the interocular ratio was zero and the probe grating was in the PE eye. The main effects of deprivation stage [*F*_(1,8)_ = 22.49, *p* < 0.001] and interocular ratio were significant [*F*_(2, 16)_ = 51.34, *p* < 0.001], but that of probe eye [*F*_(1, 8)_ = 0.01, *p* = 0.937] was not. The deprivation and probe eye interaction effect [*F*_(1, 8)_ =18.18, *p* = 0.003] was significant. LSD *post-hoc* tests showed that the perceived contrast during the deprivation stage condition differed at the PE (pre, mean ± SE = 1.06 ± 0.003; post, mean ± SE = 1.04 ± 0.003, *p* = 0.03) and the UPE (pre, mean ± SE = 1.02 ± 0.006; post, mean ± SE = 1.08 ± 0.006, *p* < 0.001). The deprivation stage and interocular contrast ratio interaction effect was significant [*F*_(2, 16)_ = 7.41, *p* = 0.005]. LSD *post-hoc* tests revealed that the perceived contrast during the deprivation stage condition was significantly different at an interocular contrast ratio of 0 (pre, mean ± SE =0.97 ± 0.003; post, mean ± SE = 1.02 ± 0.006, *p* = 0.005) but not at interocular contrast ratios of 0.8 (*p* = 0.11) and 1 (*p* = 0.06). Furthermore, the three-factor interaction was not significant [*F*_(2, 16)_ = 2.18, *p* = 0.146]. In summary, after 150 min of MPD, the perceptual eye dominance shifts to the PE.

### Modeling

In the MCM (Huang et al., 2011), the relationship between the perceived phase and contrast of the cyclopean image and the interocular contrast ratio was modeled with six parameters: (1) γ_1_ represents the non-linearity factor in the process of contrast-gain control; (2) γ_2_ represents the exponent used to control the power-law summation; (3) ρ represents the gain control efficiency of the signal strength; (4) A1 is the signal gain in the non-dominant (attenuated) eye; (5) A2, direct interocular inhibition, represents the contrast-gain control from the dominant eye to non-dominant eye; and (6) A3, indirect interocular inhibition, represents the interocular contrast-gain control from the dominant eye to the contrast-gain control signal from the non-dominant eye.

We focused on the potential involvement of one or any combination of three mechanisms (see the detail of the mathematical expression of the model in Supplementary Information)—signal gain in the PE (A1), direct interocular inhibition (A2), and indirect interocular inhibition (A3)—at two deprivation stages with a nested-model framework. We tested eight combinations of the six parameters, ranging from the full model, which assumes A1, A2, and A3 all varied after deprivation, to the most reduced model, which assumes no variation in any parameter (see Table 1).

**Table 1:** Potential models.

**Models**	**Deprivation stage**				
		**Pre**		**Post**	
Full	A1		A1	A2	A3
R1	A1		A1	A2	
R2	A1		A1		A3
R3	A1			A2	A3
R4	A1		A1		
R5	A1			A2	
R6	A1				A3
R7	A1				

*Eight potential models were developed. Because the binocular function of normal subjects at the pre-deprivation stage was still slightly unbalanced, we freed A1 to maximize the R-square fit. In addition to A1, A2, and A3, each model included three free parameters (e.g., γ_1_, ρ, and γ_2_), which were never changed by deprivation*.

Each participant's data were fitted separately using a nonlinear least-square method in MATLAB software (MathWorks, MA, US). This method was used to minimize the sum of squared error of prediction (SSE). $SSE=\sum {\left(y_{i}-{ŷ}_{i}\right)}^{2}$, where *y*_*i*_ represents the observed values and ŷ_*i*_ represents the predicted values, *y* represents the mean of all observed values. The goodness of each model was defined as *r*^2^, where:


(6) $$\begin{array}{l} r^{2}=1-\frac{\sum {\left(y_{i}-{ŷ}_{i}\right)}^{2}}{\sum {\left(y_{i}-y\right)}^{2}}, \end{array}$$

The performance of the models was evaluated by the *F* test:


(7) $$\begin{array}{l} F\left(df_{1},df_{2}\right)=\frac{\frac{\left(r_{full}^{2}-r_{reduced}^{2}\right)}{df_{1}}}{\frac{\left(1-r_{full}^{2}\right)}{df_{2}}}, \end{array}$$

where *df*_1_ = *k*_*full*_ − *k*_*reduced*_, *df*_2_ = *N* − *k*_*full*_, *k*_*full*_ is the number of parameters of the full model, *k*_*reduced*_ is the number of parameters of the reduced models, and *N* is the number of data points. The model that had the fewest parameters that remained statistically equivalent to those of the full model was used as the best-fitting model.

**Figure 2:**
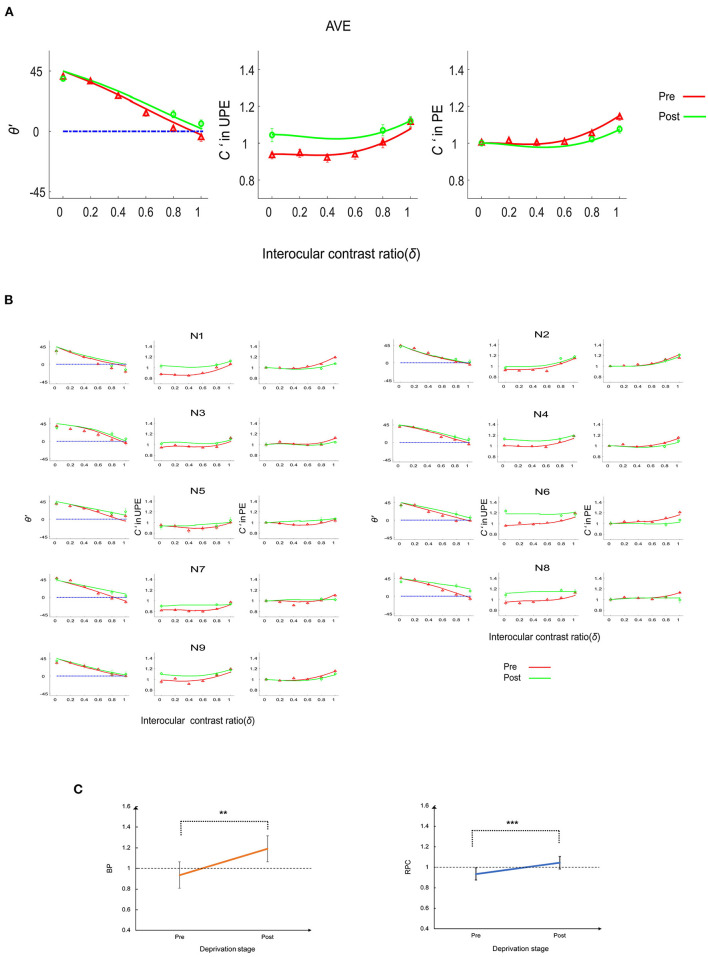
The deprivation effect on the phase and contrast co-measurement tasks. **(A)** Perceived phase and contrast for the average (AVE) of nine subjects. The first column represents the perceived phase (θ'), the second column represents the perceived contrast (*C'*) when the probe grating was displayed to the UPE, and the third column represents the perceived contrast when the probe grating was displayed to the PE. The solid lines (red and green) represent the model predictions. **(B)** Perceived phase and contrast for nine individual subjects (N1–N9). The first and fourth columns represent the perceived phase (θ'), the second and fifth columns indicate the perceived contrast (*C'*) when the probe grating was displayed to the UPE, and the third and sixth columns represent the perceived contrast when the probe grating was displayed to the PE. The solid lines (red and green) represent the model predictions. **(C)** Balance point (BP) and the ratio of perceived contrast (RPC) change. Error bars represent the SE. Asterisks indicate statistically significant effects: **p < 0.01; ***p < 0.001.

The full model, which assumed a stronger monocular signal in the PE (A1), attenuated direct interocular inhibition of the PE by the UPE (A2), and attenuated indirect interocular inhibition of the PE by the UPE (A3) after deprivation, was the best-fitting model for N7 and N8. The full model was significantly more precise than any reduced model (all *p* < 0.05). Reduced model (R1), which assumed a stronger monocular signal in the PE (A1) and attenuated interocular inhibition of the PE by the UPE (A2) after deprivation, was the best-fitting model for N2. Reduced model (R3), which assumed attenuated, direct interocular inhibition of the PE by the UPE (A2) and attenuated, indirect interocular inhibition of the PE by the UPE (A3) after deprivation was the best-fitting model for N5. The reduced model (R4), which assumed a stronger monocular signal in the PE (A1), was the best-fitting model for N1, N3, N4, N6, and N9. Furthermore, the reduced model (R4) was also the best-fitting model for the data averaged across the nine subjects (see Figure 2). According to the relevant model selection theory (Vrieze, 2012; Aho et al., 2014), we also have added the Bayesian information criterion (BIC) values of each model (see detailed analysis in Supplementary Information). The results of the model selection method using *F* analysis and BIC are consistent for nine individual subjects (N1–N9) and their average (AVE). The parameters of the best fit model are shown in Table 2.

**Table 2:** Parameters of the best-fitting model.

**Subject number**	**Best-fitting model**	**Deprivation stage**	**γ_1_**	**ρ**	**γ_2_**	**A1**	**A2**	**A3**	***r* ^2^**
N1	R4 (A1)	Pre	1.42	1.04	2.75	0.88	1.00	1.00	81.60%
		Post				1.18	1.00	1.00	
N2	R1 (A1A2)	Pre	1.72	4.92	1.49	0.92	1.00	1.00	95.62%
		Post				1.07	0.63	1.00	
N3	R4 (A1)	Pre	2.06	25.49	1.13	0.95	1.00	1.00	78.10%
		Post				1.09	1.00	1.00	
N4	R4 (A1)	Pre	1.44	8.91	1.12	1.00	1.00	1.00	94.41%
		Post				1.12	1.00	1.00	
N5	R3 (A2A3)	Pre	1.71	10.74	1.42	0.94	1.00	1.00	80.98%
		Post				1.00	0.36	0.10	
N6	R4 (A1)	Pre	1.90	5.58	1.53	0.96	1.00	1.00	86.36%
		Post				1.23	1.00	1.00	
N7	Full (A1A2A3)	Pre	1.67	14.06	1.36	0.85	1.00	1.00	94.46%
		Post				1.06	0.38	0.14	
N8	Full (A1A2A3)	Pre	2.25	17.89	1.28	0.95	1.00	1.00	82.74%
		Post				1.18	0.50	0.14	
N9	R4 (A1)	Pre	1.28	2.26	1.64	0.98	1.00	1.00	88.07%
		Post				1.11	1.00	1.00	
AVE	R4 (A1)	Pre	1.54	6.75	1.31	0.94	1.00	1.00	95.45%
		Post				1.11	1.00	1.00	

*Parameters of the best-fitting model for subjects. For each subject (N1–N9) and their average (AVE), the first and second rows represent the parameters A1, A2, and A3 for the pre-deprivation and post-deprivation periods, respectively. r^2^ represents the maximum amount of explanation for the best-fitting model*.

To better illustrate the relationship between the deprivation stage and the perceived phase and contrast, we calculated the balance point (BP) and the ratio of perceived contrast (RPC) as indexes of the deprivation-induced changes in perceptual eye dominance.

The BP, the intersection between the perceived phase vs. ratio (PVR) curve and a horizontal line (see Figure 2C) at the zero-phase point on the y axis, is a representative index of the effective contrast ratio between the PE and UPE in the phase combination task. If the BP is 1, the two eyes have equal strength in the binocular phase combination; if the BP is <1, the PE is weaker than the UPE in the binocular phase combination; if the BP is >1, the PE is stronger than the UPE in the phase combination. We replotted the interocular ratios corresponding to the BP as a function of the deprivation stage in Figure 2C. Averaged across nine subjects, the BP increased from 0.94 ± 0.02 (mean ± SE) at the pre-deprivation stage to 1.19 ± 0.06 (mean ± SE) at the post-deprivation stage. According to a paired-samples *t-*test (two-tailed), the difference in the BP was significant [*t*_(8)_ = −4.20, *p* = 0.003], indicating that 150 min of MPD induced strengthening of the PE in the binocular phase combination.

To better understand the deprivation effect on contrast matching, we calculated the RPC by dividing the normalized perceived contrast when the probe grating was displayed to the UPE by the normalized perceived contrast when the probe grating was displayed to the PE after averaging them across interocular ratio conditions (see Figure 2C). An RPC of 1 means that the two eyes were balanced in the contrast combination. If the RPC is <1, the PE is weaker than the UPE in the contrast combination. Finally, if the RPC is >1, the PE is stronger than the UPE in the contrast combination. Deprivation increased the RPC from 0.94 ± 0.02 (mean ± SE) at the pre-deprivation stage to 1.05 ± 0.03 (mean ± SE) at the post-deprivation stage; this difference was statistically significant [paired-samples *t-*test: *t*_(8)_ = −5.12, *p* = 0.001], indicating that deprivation made the PE stronger in the binocular contrast combination.

## Discussion

This current study used binocular phase and contrast combination tasks with 150 min of MPD and an MCM model to determine potential mechanisms of perceptual eye dominance plasticity. We found that a short period of MPD affects binocular function through monocular signal enhancement of the PE.

First, the current study verified the presence of remnant neuroplasticity in adult humans. After 150 min of MPD, the perceptual eye dominance shifts to the PE; in other words, the PE becomes more dominant, and the UPE becomes weaker. In recent years, an increasing number of psychophysical task studies on 150 min of monocular deprivation have reported the presence of visual neuroplasticity in adult humans. These studies, performed with transparent occlusion, found perceptual eye dominance alterations in the PE (Lunghi et al., 2011; Zhou et al., 2013a; Bai et al., 2017; Baldwin and Hess, 2018). Previous investigations have also indicated that the perceived cyclopean phase was dependent on the relative contrast ratio and phase of monocular images (Bai et al., 2017; Zhou et al., 2017a,b). However, these studies only measured one interocular contrast ratio; in the current study, we recorded six interocular ratios during the full phase and contrast co-measurement task at the pre-deprivation stage and three interocular ratios during the fast phase and contrast co-measurement task at the post-deprivation stage. Therefore, unlike in previous studies, our study was able to observe changes in the PVR curve and BP, which helped us to better understand the effect of deprivation on binocular contrast combinations.

Previous studies used binocular contrast matching tasks to assess the effect of deprivation on perceived binocular contrast (Zhou et al., 2013a, 2017a), in which the monocular perceived contrast from the PE to the UPE was used to reflect the deprivation effect. Although the authors found that the PE requires less contrast to match the perception of the UPE, they only measured one interocular contrast ratio. Therefore, these studies could not observe changes in the contrast vs. ratio (CVR) curve or the RPC. In addition to recording the effect of deprivation on phase perception, the full phase, and contrast co-measurement task can be used to assess the effect of deprivation on perceived binocular contrast in our study. To better observe and understand the effect of deprivation on contrast matching, we recorded six interocular ratios during the full phase and contrast co-measurement task at the pre-deprivation stage and three interocular ratios during the fast phase and contrast co-measurement task at the post-deprivation stage. We found that the increased UPE contrast threshold and the decreased PE contrast threshold were significant, indicating perceptual eye dominance shifts from the UPE to the PE.

The results of these psychophysical tasks suggest that the deprivation effect involves the early visual cortex; previous electrophysiology and brain imaging studies have also indicated that the deprivation effect involves the primary visual cortex (Lunghi et al., 2015a; Zhou et al., 2015; Binda et al., 2018). For instance, in one study, after 150 min of MPD, the amplitude (C1) decreased for the UPE and increased for the PE in adult humans (Lunghi et al., 2015a). Similarly, an fMRI study showed that 120 min of MPD increased the blood-oxygen-level-dependent (BOLD) response in V1 in adult humans following stimulation of the PE. However, the possible mechanisms underlying short-term MPD-induced perceptual eye dominance changes in adult humans remain unclear.

Most importantly, the primary aim of our work was to use the MCM to explain the potential mechanisms of perceptual eye dominance plasticity by 150 min of MPD. The results averaged across the nine subjects best fit the reduced model (R4). These results indicate that the deprivation effects involve the monocular signal enhancement of the PE. This study produced results which corroborate the findings of the previous work in electrophysiology. Zhou et al. (2015) have detected the underlying mechanisms of 150 min of monocular translucent patching by measuring visual evoked potentials (VEP) in early visual areas of each eye. They have found that the PE neural responses increased after deprivation, at the same time as the UPE neural responses remained the same. In the future, it is interesting to link the changes of the monocular parameter in MCM with that of VEP after MPD.

The findings of our research might have important clinical implications for amblyopia treatment. Zhou et al. (2013b) found that adults with amblyopia had a larger short-term eye deprivation effect than normal adult controls, which probably suggests that the visual cortex of adults with amblyopia has a greater degree of binocular plasticity. The findings of the present study show that the short-term monocular deprivation effect on binocular function involves a stronger monocular signal in the PE. Therefore, these findings indicated that short-term monocular (amblyopic eye) deprivation could be used to modulate unbalanced binocular function in patients with amblyopia.

To identify potential general mechanisms, further experimental investigations are needed to estimate the strengthening effect of deprivation from other physical factors (such as contrast, spatial frequency, and luminance) on the binocular function of normal and amblyopic populations.

## Data Availability Statement

The original contributions presented in the study are included in the article/Supplementary Material, further inquiries can be directed to the corresponding author/s.

## Ethics Statement

The studies involving human participants were reviewed and approved by the Ethical Review Committee of Chengde Medical University. The patients/participants provided their written informed consent to participate in this study.

## Author Contributions

JT, PZ, and ZY designed the experiment, wrote the manuscript, and collected and analyzed the data. JT, PZ, ZY, JinwL, ZC, JingL, JH, and DW revised the manuscript. All authors contributed to the article and approved the submitted version.

## Funding

This work was supported by Natural Science Foundation of Hebei Province of China (C2021205005 to PZ; C2019205282 to JH), University-Level Scientific Research Project in CDMC (202113 to JT; KY202107 to ZY), and Technology Innovation Guidance Project-Science and Technology Work Conference of Hebei Provincial Department of Science and Technology to JT.

## Conflict of Interest

The authors declare that the research was conducted in the absence of any commercial or financial relationships that could be construed as a potential conflict of interest.

## Publisher's Note

All claims expressed in this article are solely those of the authors and do not necessarily represent those of their affiliated organizations, or those of the publisher, the editors and the reviewers. Any product that may be evaluated in this article, or claim that may be made by its manufacturer, is not guaranteed or endorsed by the publisher.
