# Supplementary material for: The Mechanism of Short-Term Monocular Pattern Deprivation-Induced Perceptual Eye Dominance Plasticity
Source: Front Hum Neurosci. 2022 May 31;16:854003. doi: 10.3389/fnhum.2022.854003 (PMC9192955; doi:10.3389/fnhum.2022.854003)
Supplement: Supplementary file 1 [file Data_Sheet_1.docx]

**Text 1. The MCM of binocular combination for 150 minutes of MPD**

The MCM (Huang et al., 2010; Huang et al., 2011) elaborated on the Ding and Sperling (2006) binocular combination model. The input signals of the two monocular sine-wave gratings for binocular combination in MCM (Huang et al., 2010; Huang et al., 2011) are defined as:

$\mathrm{Lum}_{L} \left( y \right)=L_{0}\left[ 1-C_{0}\cos\left( 2\pi fy\pm\frac{\theta}{2} \right) \right]$ , (E1)

$\mathrm{Lum}_{R} \left( y \right)=L_{0}\left[ 1-\delta C_{0}\cos\left( 2\pi fy\mp\frac{\theta}{2} \right) \right]$ , (E2)

Huang et al. (2011) set base grating in the amblyopic eye (left eye, L). In the current study, we set base grating in the PE (PE represents the patched eye, UPE represents the unpatched eye), therefore PE = L and UPE =R, the input signals of the two monocular sine-wave gratings for binocular combination are defined as:

$\mathrm{Lum}_{PE} \left( y \right)=L_{0}\left[ 1-C_{0}\cos\left( 2\pi fy\pm\frac{\theta}{2} \right) \right]$ , (E3)

$\mathrm{Lum}_{UPE} \left( y \right)=L_{0}\left[ 1-\delta C_{0}\cos\left( 2\pi fy\mp\frac{\theta}{2} \right) \right]$ , (E4)

where *L*_0_ represents the value of the background in grayscale, the sine-wave grating spatial frequency is ƒ = 1 c/deg, the six interocular contrast ratios (*δ)* are 0, 0.2, 0.4, 0.6, 0.8, and 1, and the base contrast (*C*_0_) is 0.32. The two gratings, which differed by a 45° (*θ*) phase shift, were monitored by a stereoscope to produce a single fusion grating.

In MCM, the input signals of each eye first go through double interocular contrast gain control; direct interocular inhibition: each eye exerts gain control on the signal of the other eye, and indirect interocular inhibition: each eye exerts gain control over the other eye’s gain control (Huang et al., 2010; Huang et al., 2011). Therefore, the signals in the PE and UPE become:

$Lum_{PE}^{'}=\frac{1}{1+\frac{\varepsilon_{UPE}}{1+\varepsilon_{PE}}}\mathrm{Lum}_{PE}$, (E5)

$Lum_{UPE}^{'}=\frac{1}{1+\frac{\varepsilon_{PE}}{1+\varepsilon_{UPE}}}Lum_{UPE}$, (E6)

where $\varepsilon_{PE}$ and $\varepsilon_{UPE}$ are the total contrast energy (TCE) presented to the PE and the UPE, and are modelled as $\varepsilon_{PE}=\rho C_{PE}^{\gamma_{1}} \mathrm{and} \varepsilon_{UPE}=\rho C_{UPE}^{\gamma_{1}}$. *γ_1_* represents the nonlinearity factor in the process of contrast-gain control; *ρ* represents the gain control efficiency of the signal strength.

We considered three possible mechanisms of deprivation effects within the framework of the MCM: A1 represents a stronger monocular signal in the PE, A2 represents attenuate contrast gain control of the UPE on the PE (direct interocular inhibition), and A3 represents the attenuated inhibition effect from the UPE to the gain control signal from the PE (indirect interocular inhibition). The three mechanisms are modeled as modulators on the corresponding pathways in the MCM. A1, A2, and A3 are added to E5 and E6:

$Lum_{PE}^{'}=\frac{1}{1+\frac{\rho C_{UPE}^{\gamma_{1}}}{1+\rho C_{PE}^{\gamma_{1}}}}\mathrm{Lum}_{PE}=\frac{A_{1}}{1+\frac{A_{2}\rho C_{UPE}^{\gamma_{1}}}{1+{\rho\left( A_{1}C_{PE} \right)}^{\gamma_{1}}}} \mathrm{Lum}_{PE}$, (E7)

$Lum_{UPE}^{'}=\frac{1}{1+\frac{\rho C_{PE}^{\gamma_{1}}}{1+\rho C_{UPE}^{\gamma_{1}}}}{Lum}_{UPE}=\frac{1}{1+\frac{\rho\left( A_{1}C_{PE} \right)^{\gamma_{1}}}{1+\rho C_{UPE}^{\gamma_{1}}A_{3}}} {Lum}_{UPE}$ , (E8)

In the current study, *C_PE_* = *C_0_*, and *C_UPE_* = *δC_0_*. From E7 and E8, we have E9 and E10:

$Lum_{PE}^{'}=\frac{A_{1}}{1+\frac{A_{2}\rho\left( \delta C_{0} \right)^{\gamma_{1}}}{1+{\rho\left( A_{1}C_{0} \right)}^{\gamma_{1}}}}\mathrm{Lum}_{PE}=\frac{A_{1}+\rho A_{1}^{1+\gamma_{1}}C_{0}^{\gamma_{1}}}{1+\rho A_{1}^{\gamma_{1}}C_{0}^{\gamma_{1}}+A_{2}\rho\delta^{\gamma_{1}}C_{0}^{\gamma_{1}}}\mathrm{Lum}_{PE}$, (E9)

$\mathrm{Lum}_{UPE}^{'}=\frac{1}{1+\frac{\rho\left( A_{1}C_{0} \right)^{\gamma_{1}}}{1+\rho\left( \delta C_{0} \right)^{\gamma_{1}}A_{3}}}\mathrm{Lum}_{UPE}= \frac{1+\rho\delta^{\gamma_{1}}C_{0}^{\gamma_{1}}A_{3}}{1+\rho\delta^{\gamma_{1}}C_{0}^{\gamma_{1}}A_{3}+\rho A_{1}^{\gamma_{1}}C_{0}^{\gamma_{1}}}\mathrm{Lum}_{UPE}$, (E10)

The cyclopean image for the model of Ding is computed directly from the sum of $\text{ }\mathrm{Lum}_{PE}^{'}$ and $\mathrm{Lum}_{UPE}^{'}$, following Ding and Sperling (2006), E11 can be written as:

$Lum^{'}=Lum_{PE}^{'}\text{ }+Lum_{UPE}^{'}$, (E11)

Bringing E3, E4, E9, and E10 into E11, we have E12:

$Lum^{'}=\frac{A_{1}+\rho A_{1}^{1+\gamma_{1}}C_{0}^{\gamma_{1}}}{1+\rho A_{1}^{\gamma_{1}}C_{0}^{\gamma_{1}}+A_{2}\rho\delta^{\gamma_{1}}C_{0}^{\gamma_{1}}}L_{0}\left[ 1-C_{0}\cos\left( 2\pi fy\pm\frac{\theta}{2} \right) \right]$+$\frac{1+\rho\delta^{\gamma_{1}}C_{0}^{\gamma_{1}}A_{3}}{1+\rho\delta^{\gamma_{1}}C_{0}^{\gamma_{1}}A_{3}+\rho A_{1}^{\gamma_{1}}C_{0}^{\gamma_{1}}}L_{0}\left[ 1-\delta C_{0}\cos\left( 2\pi fy\mp\frac{\theta}{2} \right) \right]$

$\left. =\left[ \frac{A_{1}+\rho A_{1}^{1+\gamma_{1}}C_{0}^{\gamma_{1}}}{1+\rho A_{1}^{\gamma_{1}}C_{0}^{\gamma_{1}}+A_{2}\rho\delta^{\gamma_{1}}C_{0}^{\gamma_{1}}}+\frac{1+\rho\delta^{\gamma_{1}}C_{0}^{\gamma_{1}}A_{3}}{1+\rho\delta^{\gamma_{1}}C_{0}^{\gamma_{1}}A_{3}+\rho A_{1}^{\gamma_{1}}C_{0}^{\gamma_{1}}}-\frac{A_{1}C_{0}+\rho A_{1}^{1+\gamma_{1}}C_{0}^{1+\gamma_{1}}}{1+\rho A_{1}^{\gamma_{1}}C_{0}^{\gamma_{1}}+A_{2}\rho\delta^{\gamma_{1}}C_{0}^{\gamma_{1}}}\cos\left( 2\pi fy\pm\frac{\theta}{2} \right)-\frac{\delta C_{0}+\rho\delta^{1+\gamma_{1}}C_{0}^{1+\gamma_{1}}A_{3}}{1+\rho\delta^{\gamma_{1}}C_{0}^{\gamma_{1}}A_{3}+\rho A_{1}^{\gamma_{1}}C_{0}^{\gamma_{1}}}\cos\left( 2\pi y \right)\mp\frac{\theta}{2} \right) \right]L_{0}$,（E12）

There are two-phase shift conditions in the current study. The subscript “D” was used to represent derivations from the model of Ding (Ding and Sperling, 2006), If the phase is $\frac{\theta}{2}$ in the PE and$-\frac{\theta}{2}$in the UPE, E12 can be rewritten as:

$Lum^{'}=\left[ \frac{A_{1}+\rho A_{1}^{1+\gamma_{1}}C_{0}^{\gamma_{1}}}{1+\rho A_{1}^{\gamma_{1}}C_{0}^{\gamma_{1}}+A_{2}\rho\delta^{\gamma_{1}}C_{0}^{\gamma_{1}}}+\frac{1+\rho\delta^{\gamma_{1}}C_{0}^{\gamma_{1}}A_{3}}{1+\rho\delta^{\gamma_{1}}C_{0}^{\gamma_{1}}A_{3}+\rho A_{1}^{\gamma_{1}}C_{0}^{\gamma_{1}}}-C_{D1}^{'}\times\cos\left( 2\pi fy+\theta_{D1}^{'} \right) \right]L_{0}$, (E13)

$C_{D1}^{'}=\sqrt{\left( \frac{A_{1}C_{0}+\rho A_{1}^{1+\gamma_{1}}C_{0}^{1+\gamma_{1}}}{1+\rho A_{1}^{\gamma_{1}}C_{0}^{\gamma_{1}}+A_{2}\rho\delta^{\gamma_{1}}C_{0}^{\gamma_{1}}} \right)^{2}+\left( \frac{\delta C_{0}+\rho\delta^{1+\gamma_{1}}C_{0}^{1+\gamma_{1}}A_{3}}{1+\rho\delta^{\gamma_{1}}C_{0}^{\gamma_{1}}A_{3}+\rho A_{1}^{\gamma_{1}}C_{0}^{\gamma_{1}}} \right)^{2}+2\frac{\left( A_{1}C_{0}+\rho A_{1}^{1+\gamma_{1}}C_{0}^{1+\gamma_{1}} \right)\times\left( \delta C_{0}+\rho\delta^{1+\gamma_{1}}C_{0}^{1+\gamma_{1}}A_{3} \right)}{\left( 1+\rho A_{1}^{\gamma_{1}}C_{0}^{\gamma_{1}}+A_{2}\rho\delta^{\gamma_{1}}C_{0}^{\gamma_{1}} \right)\left( 1+\rho\delta^{\gamma_{1}}C_{0}^{\gamma_{1}}A_{3}+\rho A_{1}^{\gamma_{1}}C_{0}^{\gamma_{1}} \right)}\cos\theta}$, (E14)

When the phase is $-\frac{\theta}{2}$in the PE and $\frac{\theta}{2}$ in the UPE, E12 can be rewritten as:

$Lum^{'}=\left[ \frac{A_{1}+\rho A_{1}^{1+\gamma_{1}}C_{0}^{\gamma_{1}}}{1+\rho A_{1}^{\gamma_{1}}C_{0}^{\gamma_{1}}+A_{2}\rho\delta^{\gamma_{1}}C_{0}^{\gamma_{1}}}+\frac{1+\rho\delta^{\gamma_{1}}C_{0}^{\gamma_{1}}A_{3}}{1+\rho\delta^{\gamma_{1}}C_{0}^{\gamma_{1}}A_{3}+\rho A_{1}^{\gamma_{1}}C_{0}^{\gamma_{1}}}-C_{D2}^{'}\times\cos\left( 2\pi fy+\theta_{D2}^{'} \right) \right]L_{0}$, (E15)

$C_{D2}^{'}=\sqrt{\left( \frac{A_{1}C_{0}+\rho A_{1}^{1+\gamma_{1}}C_{0}^{1+\gamma_{1}}}{1+\rho A_{1}^{\gamma_{1}}C_{0}^{\gamma_{1}}+A_{2}\rho\delta^{\gamma_{1}}C_{0}^{\gamma_{1}}} \right)^{2}+\left( \frac{\delta C_{0}+\rho\delta^{1+\gamma_{1}}C_{0}^{1+\gamma_{1}}A_{3}}{1+\rho\delta^{\gamma_{1}}C_{0}^{\gamma_{1}}A_{3}+\rho A_{1}^{\gamma_{1}}C_{0}^{\gamma_{1}}} \right)^{2}+2\frac{\left( A_{1}C_{0}+\rho A_{1}^{1+\gamma_{1}}C_{0}^{1+\gamma_{1}} \right)\times\left( \delta C_{0}+\rho\delta^{1+\gamma_{1}}C_{0}^{1+\gamma_{1}}A_{3} \right)}{\left( 1+\rho A_{1}^{\gamma_{1}}C_{0}^{\gamma_{1}}+A_{2}\rho\delta^{\gamma_{1}}C_{0}^{\gamma_{1}} \right)\left( 1+\rho\delta^{\gamma_{1}}C_{0}^{\gamma_{1}}A_{3}+\rho A_{1}^{\gamma_{1}}C_{0}^{\gamma_{1}} \right)}\cos\theta}$ , (E16)

Following Ding and Sperling (2006), the perceived contrast of the cyclopean image is defined as:

$$\begin{aligned} C_{D}^{'}=\frac{C_{D1}^{'}+C_{D2}^{'}}{2} \\ &=\sqrt{\left( \frac{A_{1}C_{0}+\rho A_{1}^{1+\gamma_{1}}C_{0}^{1+\gamma_{1}}}{1+\rho A_{1}^{\gamma_{1}}C_{0}^{\gamma_{1}}+A_{2}\rho\delta^{\gamma_{1}}C_{0}^{\gamma_{1}}} \right)^{2}+\left( \frac{\delta C_{0}+\rho\delta^{1+\gamma_{1}}C_{0}^{1+\gamma_{1}}A_{3}}{1+\rho\delta^{\gamma_{1}}C_{0}^{\gamma_{1}}A_{3}+\rho A_{1}^{\gamma_{1}}C_{0}^{\gamma_{1}}} \right)^{2}+2\frac{\left( A_{1}C_{0}+\rho A_{1}^{1+\gamma_{1}}C_{0}^{1+\gamma_{1}} \right)\times\left( \delta C_{0}+\rho\delta^{1+\gamma_{1}}C_{0}^{1+\gamma_{1}}A_{3} \right)}{\left( 1+\rho A_{1}^{\gamma_{1}}C_{0}^{\gamma_{1}}+A_{2}\rho\delta^{\gamma_{1}}C_{0}^{\gamma_{1}} \right)\left( 1+\rho\delta^{\gamma_{1}}C_{0}^{\gamma_{1}}A_{3}+\rho A_{1}^{\gamma_{1}}C_{0}^{\gamma_{1}} \right)}\cos\theta} \end{aligned}$$

, (E17)

Furthermore, Huang et al. (2011) elaborated the Ding and Sperling (2006) model by adding an independent contrast pathway. In the new pathway, the phase information in ${Lum^{'}}_{PE}$ and ${Lum^{'}}_{UPE}$ is discarded. The contrast energies are extracted and combined to forecast the perceived contrast of the cyclopean image in the two eyes. To account for possible mechanisms of deprivation effects, following (Huang et al., 2011), the perceived contrast of the cyclopean image is defined as (*γ_2_* represents the exponent used to control the power-law summation):

$C^{'}=\left[ \left( \frac{A_{1}C_{0}+\rho A_{1}^{1+\gamma_{1}}C_{0}^{1+\gamma_{1}}}{1+\rho A_{1}^{\gamma_{1}}C_{0}^{\gamma_{1}}+A_{2}\rho\delta^{\gamma_{1}}C_{0}^{\gamma_{1}}} \right)^{\gamma_{2}}+ \right.\left. \left( \frac{\delta C_{0}+A_{3}\rho\delta^{1+\gamma_{1}}C_{0}^{1+\gamma_{1}}}{1+\rho A_{1}^{\gamma_{1}}C_{0}^{\gamma_{1}}+A_{3}\rho\delta^{\gamma_{1}}C_{0}^{\gamma_{1}}} \right)^{\gamma_{2}} \right]^{\frac{1}{\gamma_{2}}}$, (E18)

From E9 and E10, the double interocular contrast gain control (DB) for each eye has been figured out:

${DB}_{PE}=\frac{A_{1}+\rho A_{1}^{1+\gamma_{1}}C_{0}^{\gamma_{1}}}{1+\rho A_{1}^{\gamma_{1}}C_{0}^{\gamma_{1}}+A_{2}\rho\delta^{\gamma_{1}}C_{0}^{\gamma_{1}}}$, (E19)

${DB}_{UPE}=\frac{1+\rho\delta^{\gamma_{1}}C_{0}^{\gamma_{1}}A_{3}}{1+\rho\delta^{\gamma_{1}}C_{0}^{\gamma_{1}}A_{3}+\rho A_{1}^{\gamma_{1}}C_{0}^{\gamma_{1}}}$, (E20)

According to Ding and Sperling (2006), and Huang et al. (2011), the perceived phase in the PE and the UPE are defined as:

$\theta_{PE}^{'}=\tan^{-1} \left( \frac{{DB}_{PE}-{DB}_{UPE}}{{DB}_{PE}+{DB}_{UPE}}\tan\left( \frac{\theta}{2} \right) \right)$ , (E21)

$\theta_{UPE}^{'}=-\tan^{-1} \left( \frac{{DB}_{PE}-{DB}_{UPE}}{{DB}_{PE}+{DB}_{UPE}}\tan\left( \frac{\theta}{2} \right) \right)$, (E22)

According to Ding and Sperling (2006), and Huang et al. (2011), the perceived phase difference between the two configurations is:

$\theta^{'}=\theta_{PE}^{'}-\theta_{UPE}^{'}=2\tan^{-1} \left( \frac{{DB}_{PE}-{DB}_{UPE}}{{DB}_{PE}+{DB}_{UPE}}\tan\left( \frac{\theta}{2} \right) \right)$ , (E23)

Expanding E23, we have E24:

$\theta^{'}=2\tan^{-1} \left[ \frac{\frac{A_{1}C_{0}+\rho A_{1}^{1+\gamma_{1}}C_{0}^{1+\gamma_{1}}}{1+\rho A_{1}^{\gamma_{1}}C_{0}^{\gamma_{1}}+A_{2}\rho\delta^{\gamma_{1}}C_{0}^{\gamma_{1}}}-\frac{\delta C_{0}+A_{3}\rho\delta^{1+\gamma_{1}}C_{0}^{1+\gamma_{1}}}{1+\rho A_{1}^{\gamma_{1}}C_{0}^{\gamma_{1}}+A_{3}\rho\delta^{\gamma_{1}}C_{0}^{\gamma_{1}}}}{\frac{A_{1}C_{0}+\rho A_{1}^{1+\gamma_{1}}C_{0}^{1+\gamma_{1}}}{1+\rho A_{1}^{\gamma_{1}}C_{0}^{\gamma_{1}}+A_{2}\rho\delta^{\gamma_{1}}C_{0}^{\gamma_{1}}}+\frac{\delta C_{0}+A_{3}\rho\delta^{1+\gamma_{1}}C_{0}^{1+\gamma_{1}}}{1+\rho A_{1}^{\gamma_{1}}C_{0}^{\gamma_{1}}+A_{3}\rho\delta^{\gamma_{1}}C_{0}^{\gamma_{1}}}}\tan\left( \frac{\theta}{2} \right) \right]$,（E24）
